# Sociodemographic and Health-Service Precursors of Local-Stage Lung Cancer Diagnosis: A Population-Based Study in New South Wales, Australia

**DOI:** 10.3390/cancers17111791

**Published:** 2025-05-27

**Authors:** David Banham, David Roder, Anh-Minh Nguyen, Emily Stone, Shelley Rushton, Tracey O’Brien

**Affiliations:** 1 Cancer Institute NSW, Sydney, NSW 2065, Australia; david.banham8121@outlook.com (D.B.); shelley.rushton@health.nsw.gov.au (S.R.); tracey.obrien@health.nsw.gov.au (T.O.); 2 Cancer Epidemiology and Population Health, University of South Australia, Adelaide, SA 5001, Australia; anhminh.nguyen@unisa.edu.au; 3 Medicine & Health School of Clinical Medicine, University of NSW, Sydney, NSW 2033, Australia

**Keywords:** lung cancer stage disparities, primary care, health factors, sociodemographic predictors

## Abstract

**Aims:** To investigate sociodemographic associations with medicated health conditions, general practitioner (GP) contacts, and computed tomography (CT) scans of the lung, as 12-month precursors of diagnosis of lung cancer at a local stage (cancers localized to the primary site of bronchus and lung). **Methods:** Cancer Registry data for New South Wales (NSW) adults diagnosed with lung cancer (ICD-10 C33-34) since the Census of August 2016 (n = 6160) were linked at person level with census and other administrative data. These included residents diagnosed with lung cancer from September 2016 to December 2018. Structural equation modelling indicated adjusted measures of associations with lung cancer, including adjusted odds ratios (aORs), in stepped analyses. **Results:** The first part of the multivariate modelling showed age, major city residence, and other sociodemographic characteristics that were associated with numbers of medicated conditions. The second part showed the numbers of medicated conditions and other sociodemographic characteristics that were associated with the number of GP consultations. The third part of the modelling showed the numbers of GP consultations and other sociodemographic characteristics that were associated with having CT lung scans. Modelling showed that having CT scans and being female were the main predictors of lung cancer diagnosis at a local stage, with aORs of 2.30, 95%CI 2.01–2.63 and 1.39, and 95%CI 1.23–1.58, respectively. The modelling also showed age, GP consultations, residence in a major city, and other sociodemographic characteristics to be associated with having CT scans. **Conclusions:** The findings of the study indicate the main precursors of lung cancer diagnosis at a local stage after multivariate adjustment. Irrespective of causal significance, results reveal population-wide characteristics for targeting screening for early detection. They demonstrate the potential value of person-level linkage of cancer-registry data with census and other administrative data for this purpose. Our study of linked cancer-registry and census data revealed broad descriptive features of pathways to early diagnosis of relevance to service screening and planning.

## Simple Summary

Lung cancer is a major health problem. It ranks fifth among cancers most commonly reported to Australian cancer registries and is the leading cause of cancer death in Australia. Five-year relative survival for lung cancer is about 68% for local stage at diagnosis compared with less than 5% for metastatic stage. Diagnosis at an early stage improves survival, but only about 20% of lung cancers were found at a local stage. The aim of this study was to examine the socio-demographic associations with medicated health conditions, general practitioner consultations, and computed tomography (CT) scans of the lung as precursor predictors to lung cancer diagnosis at a local stage. Multivariate structural equation modelling suggested CT scans of the lung and being female were main predictors. This study shows the breadth of evidence that can be obtained from linking person-level data population-wide. By identifying at-risk population subgroups, the research can inform the design and implementation of lung cancer screening initiatives for maximum benefit and optimal cost-effectiveness.

## 1. Introduction

Lung cancer is the leading cause of cancer death in Australia, as it is globally [1,2]. Around 80% of cases are attributed to tobacco smoking, so primary prevention is largely focused on reducing smoking prevalence [1]. There is evidence from the USA and Europe that lung cancer screening directed at detection at an early stage can reduce lung cancer mortality [3,4]. Australian research shows markedly higher lung cancer survival when these cancers are found early [5]. The National Lung Cancer Screening Program (NLCSP) is scheduled to begin in Australia in mid-2025. It will be risk-based to optimize cost-effectiveness and the benefit-to-harm ratio, using age and smoking behavior as risk indicators [6]. It is important that due emphasis be given to promoting screening in population demographics where early detection is least common. The present study aims to identify sociodemographic and health characteristics universally covered by the census and other population-wide data that are predictive of early as opposed to later stages of lung cancer at diagnosis. Health administrations seek these data for priority setting in the planning and monitoring of early-detection initiatives.

This study is an important first step in using relevant population-wide NSW cancer registry data linked to the Australian Bureau of Statistics (ABS) Census for the purposes of early detection, monitoring, and planning. Lung cancer is a major health problem ranked in the top five diseases of the global burden of disease [2]. In Australia, lung cancer ranks fifth among cancers most commonly reported to cancer registries and is the leading cause of cancer death [1,2]. Mostly, this reflects early deaths [1]. Lung cancer outcomes are poor, with a five-year relative survival in 2016–2020 of around 26%. This compares with the 71% survival reported for all cancers combined over that period [1].

The potential for survival gains arises from early detection at a local stage [5,7,8]. In Australia, Cancer Registry data show that fewer than 17% of lung cancers are detected at a local stage, providing scope for improved outcomes through earlier detection [5]. The five-year relative survival for lung cancer is about 68% for local stage at diagnosis compared with less than 5% for metastatic stage [5].

Percentages of lung cancers diagnosed at a local stage vary, with lower percentages applying in rural areas [9,10]. Stage distributions also can vary by ancestry and ethnicity [11]. A recent meta-analysis did not find a difference in the likelihood of early-compared with later-stage lung cancer by sociodemographic disadvantage [12].

A recent study of older Australians explored pathways to local stage at lung cancer diagnosis by socioeconomic disadvantage, number of health conditions, and frequency of contact with a general practitioner (GP), respectively [13]. Results indicated socioeconomic disadvantage, comorbid health conditions, and frequency of GP contact to be associated with cancer stage at diagnosis [13].

The opportunity now presents itself to explore associations of stage with survival more fully, using comprehensive person-level data on sociodemographic, health, and health-service characteristics [14]. This study explores the utility of these data for identifying population subgroups for targeting early-detection initiatives [15,16]. We have investigated associations of sociodemographic and related factors with medicated health conditions, contacts with general practitioners (GPs), and CT lung scans as potential predictors of lung cancer diagnosis at a local (i.e., cancers localized to the primary site of bronchus and lung) compared with more advanced stage (cancers of this primary site with signs of spread to nearby lymph nodes or distant parts of the body).

## 2. Materials and Methods

### 2.1. Study Design

Participants included in the study were all New South Wales (NSW) adults aged 18 years and older since the Census of August 2016, who subsequently experienced the first diagnosis of lung cancer (ICD-10 C33-34) in the period of September 2016 to December 2018 (n = 6160). Wider descriptive contextual information on the population of NSW adults, cancer diagnoses, and epidemiological methods is reported elsewhere [17,18].

A retrospective cohort study design was used, including New South Wales (NSW) population-based registry data on lung cancer linked to extracts from the Australian Census and other administrative health data available through the Australian Bureau of Statistics (ABS) Person Level Integrated Data Asset (PLIDA) [19,20]. The PLIDA includes a unique Person Linkage Spine (PLS) for people recorded on the Australian Medicare Consumer Directory, Centrelink, or Taxation datasets in the period between 2006 and 2016. The PLS enables the ABS, as an accredited integrating authority, to link multiple datasets while protecting privacy. The present study included records of all adults aged 18 years or more living in NSW at the time of the August 2016 census, recorded on the PLS. The first invasive cancer diagnosed in the trachea or lung (ICD-10 C33-34) was included for each person for the period from September 2016 to December 2018.

### 2.2. Data Sources

Data sources included the NSW Cancer Registry (NSWCR), the Australian Census 2016, and claims made to the Pharmaceutical Benefits Scheme (PBS) and Medicare Benefits Schedule (MBS) as components of Australia’s universal health insurance scheme.

The NSW Cancer Registry provided population-based incidence data for lung cancer and stage (extent of disease) at diagnosis (local, regional, and distant/unknown stage). Census records provided sociodemographic data on age, sex, geographic remoteness using the Accessibility and Remoteness Index of Australia (ARIA), country of birth, and household composition. Census data indicated each person’s socioeconomic status of residential area, as classified by the ABS Index of Relative Socio-Economic Disadvantage (IRSD) [21], while allowing deconstruction of the Index into its component parts at the person and household level. These components are shown in the Results section (Tables S1–S4). Consistent with earlier Australian research, PBS claims were used for each person for the 12-month period preceding lung cancer diagnosis [22,23]. This was expressed using the Anatomical Therapeutic Classification (ATC) of prescribed medications, enabling the categorization of medicated conditions across the range of the Rx risk comorbidity index [23,24]. MBS records for the 12 months preceding diagnosis were used to enumerate GP consultations (a professional attendance to a “GP” or “General Practitioner” and use of computerized tomography (CT) scans of the lung (MBS diagnostic imaging items 56301; 56307; 56341; and 56347)) [25].

To avoid selection bias, data for *all* lung cancers in NSW that met the selection criteria were linked to the Census, PBS, MBS universal health insurance data, and related administrative databases.

### 2.3. Variables

The principal outcome variable was diagnosis at a local rather than a more advanced stage. The NSWCR had recorded summary staging information at diagnosis, which was dichotomized into local (=1) or more advanced spread (regional, distant, and unknown = 0). Unknown and distant spread were combined because their associated survival proportions were similar [4], and to maximize statistical precision.

Three main predictor variables were investigated along the pathway to cancer diagnosis. These predictors were based on prior research [13], including a study from the National Lung Screening Trial Research Team on lung cancers potentially being detected early through CT lung scans [3,4]. The first was the number of medicated health conditions as included in the Rx risk comorbidity index [23,24]. Using condition counts as a guide, we arranged these numbers into three groups of 0–2, 3–5, and 6 or more. We similarly grouped the second predictor (number of GP consultations) as 0–7, 8–16, and 17 or more [13]. The final predictor was having a CT scan of the lung, classified on a dichotomous scale as having a CT scan (=1) or not having a CT scan (=0).

Sociodemographic covariates included age at the census, categorized for descriptive display and expressed as a continuous measure for multivariate modelling. These covariates also included geographic remoteness of residence (major city, inner regional, or outer regional/remote); countries of birth, classified as Australia, China, Greece, Italy, Lebanon, New Zealand, the Philippines, the United Kingdom, Vietnam, “other mainly English speaking”, and “other mainly non-English speaking countries” [26]; and whether living in a lone occupant household.

Socioeconomic disadvantage (IRSD) was also included as a covariate, dichotomized using principal components and ABS methods [21,27]. These components included English language proficiency (self-reporting that English is not spoken well or not at all), low household income (household income reported as less than AUD 26,000 (equivalized using modified OECD scaling from the Organisation for Economic Co-operation and Development (https://www.oecd.org/en.html, accessed on 5 May 2025)), core function-limiting disability (self-reporting a need for assistance with core activities of self-care, mobility, or communication, due to a long-term health condition or disability), employment status and occupation (e.g., drivers, laborers, and service providers), educational attainment, residential household with children, resident parent numbers, and whether renting through a housing authority.

Data on overcrowding, household internet connection, and cars, as used in the IRSD, were not available for analysis.

### 2.4. Statistical Analysis

Bivariate analyses of local stage and the three predictor variables were investigated initially in cross-tabulations with each sociodemographic and health variable. Initial indications of associations were inferred from “*p* values” < 0.05. Separate multivariable models for the principal and other predictor variables were undertaken using least-squares linear models for those measured on a continuous scale and logistic models for CT scans as a binary variable. Local stage as a binary outcome variable was also analyzed by logistic regression. All potential covariates related to the main variables at a bivariate level (*p* < 0.10) were simultaneously evaluated in our models. Variables were purposefully removed stepwise when they did not contribute to statistically significant associations in the structural equation modelling (i.e., *p* ≥ 0.05). We refitted models with remaining covariates until a main effects model was derived, where each retained covariate contributed to model fit. Our structural modelling included directional relationships based on empirical evidence of numbers of medicated health conditions, GP contacts, and CT scanning as potential predictors of the likelihood of diagnosis at a local stage [18,28].

The potential for collinearity among covariates was tested using variance inflation factors and was not found to apply. Data preparation and analyses were implemented, using Stata 18, by remote access to de-identified data stored in the ABS Data Lab (a secure access environment).

This study was mostly conducted in 2023 and 2024 following approval by the NSW Population and Health Services Research Ethics Committee (PHSREC 2019/ETH13324).

## 3. Results

### 3.1. Medicated Health Conditions

Table S1 outlines unadjusted bivariate distributions of sociodemographic and health characteristics of cohort members across the cohort, both in total and by number of medicated health conditions. Higher numbers of medicated conditions applied to older lung cancer cases; those residing outside of major cities and more remotely; those living in sole person households; those with low incomes; younger people with a disability; those obtaining less than year 12 of schooling; those renting from a housing authority; and those having higher numbers of GP consults. (*p* ≤ 0.005). Conversely, lower numbers of medicated conditions applied to lung cancer cases born in mostly non-English speaking countries; and to laborers, machine operators, and drivers (*p* < 0.001). No associations of the number of medicated health conditions applied by sex; according to English-speaking proficiency; as receiving no education; being jobless with children; or being a single parent; and whether lung cancer was diagnosed at a local compared with a more advanced stage (*p* ≥ 0.087).

### 3.2. General Practitioner (GP) Consultations

Table S2 outlines unadjusted bivariate distributions of sociodemographic disadvantage and health characteristics with the number of GP consultations. Higher numbers of consultations applied to older lung cancer cases; those residing in major cities; those born in Greece, Italy, Lebanon, the Philippines, or other non-English speaking countries; those with poor English-speaking proficiency; those of low income status; young people with a disability; those receiving less than a year12 education; those having no education; those renting from a housing authority; those reporting a higher number of medicated health conditions; and those diagnosed with a local stage (*p* ≤ 0.005). Conversely, lower numbers of GP consultations applied to lung cancer cases who were unemployed or were laborers or machine operators/drivers at time of the census (*p* ≤ 0.009), and no associations applied of numbers of GP consultations with those living in sole person households; jobless households with children; or one-parent households with dependents (*p* ≥ 0.166).

### 3.3. Computed Tomography (CT) Scans of the Lung

Bivariate unadjusted associations of MBS-funded CT scans of the lung in the 12 months preceding diagnosis were found with sociodemographic and health characteristics (Table S3). Scans were less common among those living outside of major cities; in sole-person households; those who were younger and living with a disability; machine operators and drivers; those who had not completed year 12 of education; those who had received no formal education; those renting through a public housing authority; those living in a jobless household with children; and those having fewer GP consultations (*p* ≤ 0.020). The Australian-born tended to have CT scans less commonly, with considerable heterogeneity applying for other countries of birth (i.e., CT scans tended to be less common for those born in the Philippines and the UK, and conversely, more common in those diagnosed at a local stage and those born in China, Italy, Lebanon, Vietnam, other non-English speaking countries, or New Zealand (*p* ≤ 0.005)). No associations were found with the number of medicated health conditions; age; sex; poor English proficiency; low-income status; being unemployed; being a laborer; or being a single parent with dependents (*p* ≥ 0.064).

### 3.4. Local Stage

Bivariate unadjusted associations of local stage at diagnosis were found with sociodemographic and health characteristics (Table S4). Local stage was more common in females than males and in those having more GP consultations (*p* < 0.001). Conversely, low-stage lung cancer was less likely in those living in a jobless household with children (*p* < 0.001). Other characteristics were not associated with local stage (*p* ≥ 0.125) (Table S4).

### 3.5. Lung Cancer Diagnosis at Local Stage

**Figure 1:**
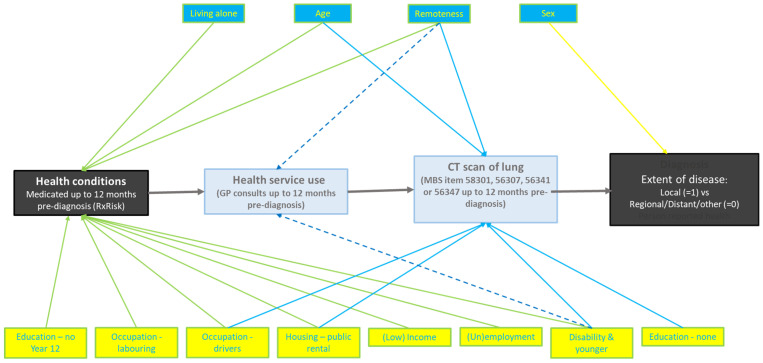
Structural pathway to the local stage.

Multivariate structural equation modelling indicated associations of local stage with variables categorized as numbers of medicated health conditions, GP consultations, and lung CT scans. The final model in Table 1, fully adjusted for these variables, retained only being female and having CT scans as predictors of local stage, the respective adjusted odds ratios being aOR 1.39, 95%CI 1.23, 1.58 and aOR 2.30, 95%CI 2.01, 2.63. The structural pathway leading to these results is depicted in Figure 1.

The structural pathway to the local stage at diagnosis, as indicated by the multivariate model, is as shown in Figure 1.

## 4. Discussion

Results indicate sociodemographic and health variables found in structural equation modelling to be associated with medicated health conditions, contact with General Practitioners, and having CT scans, respectively, as 12-month precursor predictors of lung cancer diagnosis at a local stage. After adjusting for these variables, being female and having a CT scan in the 12-month period preceding diagnosis were key predictors of diagnosis at a local stage. We consider this finding of females having a higher likelihood of presenting with a local cancer stage to be most likely a result of more frequent health care-seeking behavior for both physical and mental health issues than for males [29]. This difference by sex in health care-seeking behavior was confirmed by a study conducted in Canada using the international Quality and Cost of Primary Care (QUALICOPC) survey [30]. In Australia, females were more likely to seek their GP for symptoms or routine checkups than males [29]. In 2023–2024, 88% of females were found to have seen a GP compared to their male counterparts (80%); females also received more Medicare-subsidized GP attendances per person (7.1, compared with 5.2 for males). This pattern has been consistent since 2017–2018. In addition, females were more likely to see an allied health professional than males (44% versus 33%) and to receive more Medicare-subsidized services per person (1.2 versus 0.8). This pattern has been consistent since 2017–2018 [29].

Only one in five persons with lung cancer in this study was diagnosed at a local stage. The adjusted odds of local stage indicated by the structural equation modelling were consistent with earlier study findings, with sociodemographic factors such as geographic residence and ethnicity being associated with diagnosis at a local stage [9,10,11].

The pathway to a local-stage diagnosis was explored in multivariate analyses across three steps. The modelling indicated that the number of medicated health conditions increased with age, low income, residence outside a major city, low educational level, renting from a housing authority, and in younger adults with a disability. These attributes were likely associated with levels of socioeconomic deprivation and/or reduced access to prevention or curative care. By comparison, it was less apparent why fewer medicated conditions applied to those in sole-person households, laborers, machine operators, and the unemployed. Further research is needed to explore factors underlying these associations, including lifestyle factors and potentially limited access to services due to disabilities.

Further modelling indicated that higher numbers of GP consultations were related to higher numbers of medicated conditions, living in a major city, and being a younger adult with a disability. It is plausible that these factors would reflect access and contact with GP services. Younger adults with disabilities may require an increased use of GP services, depending on the type and severity of their disabilities. Again, further research is needed into these and alternative explanations for these findings.

The next step in the modelling indicated CT scans of the lung to be associated with younger age, living in major cities, and having higher numbers of GP consultations. Plausible explanations include increased access to and utilization of GP and specialist services. Factors negatively related to CT scanning included renting from a housing authority and receipt of little or no education, which likely reflected lower socioeconomic status. It is not clear why other negatively correlated factors presented, such as employment as a driver or machine operator, and being a younger adult with a disability. Again, further research is needed into explanations for these results.

In the fully adjusted model, the odds of local stage as the main outcome of this study were found to be 39% higher in females than males and 130% higher in those having CT scans of the lung in the 12 months preceding diagnosis.

This study has described factors associated with early diagnosis. It is descriptive and sets the stage for more in-depth research. Australia is introducing lung cancer screening, which presents challenges for how to target this screening for maximum benefit and cost-effectiveness. The present study illustrates the use of population-wide available census and health-related data of relevance for broad targeting of screening at a population level. Tobacco smoking data were not available population-wide, but it would be important to filter screening selection at the person level under operational conditions.

*Study strengths* included the use of objective data on lung cancer and lung cancer risk factors from the NSW population-based cancer registry, the census, and Pharmaceutical Benefits (PBS) and Medicare Benefits (MBS) claims for exploring predictors of steps along the lung cancer pathway to diagnosis at a local stage. The use of linked data enabled investigation of these steps in a sociodemographic context. We consider the use of these population-wide linked data at the person level to be relatively novel for Australia and to provide more reliable evidence than data from self-reporting or ecological studies. Protection of privacy was supported by legislation, use of privacy-protecting protocols by a nationally credential data linkage facility, and the storage of data for analysis in deidentified form and in a secure access environment, with analyses undertaken through remote access with independent vetting of results prior to release.

*Study limitations* included the use of dichotomized variables to facilitate analyses. Although this process was consistent with the prior use of these variables in a validated disadvantage index [21,27], dichotomizing may have reduced validity and statistical precision [31]. Other limitations included the lack of data on smoking [32] and overcrowding, as included in the SEIFA IRSD [21]. Other limitations were the exclusion of some potentially important socio-demographic markers such as internet access and private motor vehicle availability [21]. Also, the linked administrative data did not include direct measures of comorbid conditions, with reliance placed instead on indirect indicators of comorbidity from health insurance claims [33].

*Comparisons and interpretations* align with earlier studies, indicating that comorbidities recorded in hospital records often commenced prior to lung cancer diagnosis [34,35,36]. The number of medicated conditions was found to increase with increasing age, living outside major cities, having a low income, being younger with a disability, not having year-12 schooling, and renting from a housing authority. These characteristics were consistent with expectations that older people and those of lower socioeconomic status would have more medicated health conditions. Area socioeconomic disadvantage was previously found to be strongly related to lung cancer incidence in NSW and more widely [26,37,38]. However, the presence of fewer medicated conditions observed in this study in members of sole-person households, the unemployed, laborers, and machine operators, was unexpected. Potentially, this could reflect differences in intensity of care and data ascertainment.

Younger adults with disabilities were less likely, in the multivariate analysis, to have fewer medicated conditions, fewer GP consultations, and fewer CT scans. The potential for disparities in lung cancer by disability status is poorly understood [39,40]. Further study is needed into the effects of different types of mobility, sensory, learning, and cognitive limitations [40], and their association with cancer diagnosis.

Poor housing is widely acknowledged to impose health risks [41]. Housing authority rental was associated in this study with more medicated conditions and fewer CT scans. The underlying reasons for these findings require additional research, including the potential for sub-optimal tenure to contribute to poor health.

The results of this study may help guide preventive efforts toward previously under-recognized groups of people with the potential to benefit from health-related information and proportionately greater attention to their health, social, and economic needs [41,42]. Results may similarly guide the targeting of screening and use of CT scans.

## 5. Conclusions

This study shows the breadth of evidence that can be obtained from linking person-level data population-wide to describe precursor predictors of lung cancer detection at an early rather than later stage. The structural pathway used was plausible. Results indicate demographic and health characteristics of relevance to targeting of screening and other early-detection interventions at a population level. This methodology may be useful for other cancer types and chronic diseases.

## Figures and Tables

**Table 1:** Multivariate structural equation model of sociodemographic and health factors associated with the local stage of lung cancer at diagnosis *.

	β Coefficient	L95%CI	U95%CI	*p*
Medicated condition numbers *				
Age (years)	0.06	0.06	0.07	<0.001
Remoteness				
Major cities and others	0.00	Reference		
Inner regional	0.23	0.12	0.35	<0.001
Outer regional/remote	0.19	0.02	0.37	0.029
Sole-person household	−0.16	−0.28	−0.04	0.010
Low income	0.12	0.01	0.23	0.039
Younger with disability	1.18	0.96	1.39	<0.001
Laborer	−0.81	−1.10	−0.52	<0.001
Machinery operator or driver	−0.47	−0.81	−0.14	0.005
Unemployed	−0.64	−1.02	−0.26	0.001
Less than year12 schooling attained	0.19	0.09	0.29	<0.001
Renting from housing authority	0.44	0.25	0.62	<0.001
GP consult numbers **				
Remoteness				
Major cities and others	0.00	Reference		
Inner regional	−1.94	−2.45	−1.42	<0.001
Outer regional/remote	−1.97	−2.72	−1.22	<0.001
Younger with disability—	1.74	0.83	2.66	<0.001
Medicated conditions				
0–2 conditions	0.00	Reference		
3–5 conditions	3.71	3.15	4.28	<0.001
6 or more conditions	9.11	8.55	9.68	<0.001
	**adjusted Odds Ratio**	**L95%CI**	**U95%CI**	***p***
Lung CT scan ***				
Age (years)	0.99	0.99	1.00	<0.001
Remoteness				
Major cities	1.00	Reference		
Regional/remote	0.84	0.78	0.87	0.002
Younger with disability	0.62	0.49	0.77	<0.001
Driver	0.63	0.44	0.89	0.009
No education	0.61	0.42	0.88	0.014
Housing authority rental	0.72	0.76	0.86	<0.001
GP consult numbers				
0–7	1.00	Reference		
8–16	1.77	1.57	2.00	
17 or more	2.47	2.15	2.84	<0.001
Local-stage disease ****				
Lung CT scan	2.30	2.01	2.63	<0.001
Sex				
Males	1.00	Reference		
Females	1.39	1.23	1.58	<0.001

* R^2^ = 13.9%. ** R^2^ = 15.9%. *** Goodness of fit *X*^2^(8) = 12.7, *p* = 0.124, 59.3% Correctly classified. **** Goodness of fit *X*^2^(4) = 0.98, *p* = 0.982, 79.7% Correctly classified.

## Data Availability

Original data for this study were provided by the Cancer Institute of New South Wales, the Australian Bureau of Statistics, and the Australian Department of Health, with ethics committee approval. These data may be available to other researchers who meet data access and ethical requirements. Requests and enquiries regarding the data processing and analysis code for this article can be made to the lead author.

## Supplementary Materials

The following supporting information can be downloaded at: https://www.mdpi.com/article/10.3390/cancers17111791/s1, Table S1: Sociodemographic and health characteristics by numbers of medicated health conditions among adults diagnosed with lung cancer; Table S2: Sociodemographic and health characteristics by numbers of general practitioner (GP) consultations among adults diagnosed with lung cancer; Table S3: Sociodemographic and health characteristics by exposure to MBS-funded lung CT scans among adults diagnosed with lung cancer; Table S4: Sociodemographic and health characteristics by local stage at diagnosis among adults diagnosed with lung cancer.
