# Supplementary material for: Sociodemographic and Health-Service Precursors of Local-Stage Lung Cancer Diagnosis: A Population-Based Study in New South Wales, Australia
Source: Cancers (Basel). 2025 May 27;17(11):1791. doi: 10.3390/cancers17111791 (PMC12153752; doi:10.3390/cancers17111791)
Supplement: Supplementary file 1 [file cancers-17-01791-s001.zip › cancers-3588501-supplementary.pdf]

Supplementary Tables

Table S1. Sociodemographic and health characteristics by numbers of medicated health conditions among adults diagnosed with lung cancer*

				Medicated health conditions						
				0-2		3-5		6 or more		
		Total	Column %	n	Column %	n	Column %	n	Column %	p
Total		6,160	100.0%	1,487	100.0%	2,337	100.0%	2,336	100.0%	
**Sociodemographic factors**										
Mean age (years)	n (95%CI)	69.1 (68.8-69.3)		63.7 (63.1-64.3)		68.9 (68.5-69.4)		72.5 (72.2-73.0)		<0.001
Sex										
	Males	3,276	53.2%	813	54.7%	1,244	53.2%	1,219	52.2%	0.322
	Females	2,884	46.8%	674	45.3%	1,093	46.8%	1,117	47.8%	
Geographic remoteness										
	Major cities & others	4,120	66.9%	1,036	69.7%	1,584	67.8%	1,500	64.2%	0.005
	Inner regional	1,463	23.8%	315	21.2%	540	23.1%	608	26.0%	
	Outer regional	577	9.4%	136	9.1%	213	9.1%	228	9.8%	
Country of birth										
	Australia	3,749	60.9%	861	57.9%	1,411	60.3%	1,477	63.2%	<0.001
	China	176	2.9%	90	6.1%	58	2.5%	28	1.2%	
	Greece	78	1.3%	12	0.8%	27	1.2%	39	1.7%	
	Italy	124	2.0%	19	1.3%	58	2.5%	47	2.0%	
	Lebanon	79	1.3%	14	0.9%	24	1.0%	41	1.8%	
	New Zealand	129	2.1%	46	3.1%	52	2.2%	31	1.3%	
	the Philippines	47	0.8%	15	1.0%	21	0.9%	11	0.5%	
	United Kingdom	553	9.0%	117	7.9%	217	9.3%	219	9.4%	
	Vietnam	67	1.1%	21	1.4%	30	1.3%	16	0.7%	
	Other English-speaking countries	216	3.5%	53	3.6%	91	3.9%	72	3.1%	
	Other non-English speaking countries	942	15.3%	239	16.1%	348	14.9%	355	15.2%	
Sole person household										
	Yes	1,573	25.5%	332	22.3%	624	26.7%	617	26.4%	0.005

	No	4,587	74.5%	1,155	77.7%	1,713	73.3%	1,719	73.6%	
**Socioeconomic factors**										
Poor English proficiency										
	Yes	426	6.9%	111	7.5%	153	6.5%	162	6.9%	0.551
	No	5,734	93.1%	1,376	92.5%	2,184	93.5%	2,174	93.1%	
Low income										
	Yes	2,059	33.4%	400	26.9%	783	33.5%	876	37.5%	<0.001
	No	4,101	66.6%	1,087	73.1%	1,554	66.5%	1,460	62.5%	
Younger with disability										
	Yes	366	5.9%	47	3.2%	121	5.2%	198	8.5%	<0.001
	No	5,794	94.1%	1,440	96.8%	2,216	94.8%	2,138	91.5%	
Unemployed										
	Yes	109	1.8%	54	3.6%	42	1.8%	13	0.6%	<0.001
	No	6,051	98.2%	1,433	96.4%	2,295	98.2%	2,323	99.4%	
Labourer										
	Yes	194	3.1%	93	6.3%	77	3.3%	24	1.0%	<0.001
	No	5,966	96.9%	1,394	93.7%	2,260	96.7%	2,312	99.0%	
Machinery operator & driver										
	Yes	143	2.3%	60	4.0%	57	2.4%	26	1.1%	<0.001
	No	6,017	97.7%	1,427	96.0%	2,280	97.6%	2,310	98.9%	
Less than Year 12 schooling attained										
	Yes	2,855	46.3%	606	40.8%	1,056	45.2%	1,193	51.1%	<0.001
	No	3,305	53.7%	881	59.2%	1,281	54.8%	1,143	48.9%	
No education										
	Yes	129	2.1%	27	1.8%	45	1.9%	57	2.4%	0.325
	No	6,031	97.9%	1,460	98.2%	2,292	98.1%	2,279	97.6%	
Renting from housing authority										
	Yes	542	8.8%	91	6.1%	201	8.6%	250	10.7%	<0.001
	No	5,618	91.2%	1,396	93.9%	2,136	91.4%	2,086	89.3%	
Jobless household with children										
	Yes	98	1.6%	32	2.2%	34	1.5%	32	1.4%	0.136
	No	6,062	98.4%	1,455	97.8%	2,303	98.5%	2,304	98.6%	
One parent with dependents										
	Yes	102	1.7%	33	2.2%	39	1.7%	30	1.3%	0.087
	No	6,058	98.3%	1,454	97.8%	2,298	98.3%	2,306	98.7%	

**Health factors**										
GP consults										
0-7	1,852	30.1%	832	56.0%	709	30.3%	311	13.3%	<0.001
8-16	2,651	43.0%	521	35.0%	1,160	49.6%	970	41.5%	
17 or more	1,657	26.9%	134	9.0%	468	20.0%	1,055	45.2%	
Local stage at diagnosis									
Yes	1,253	20.3%	292	19.6%	464	19.9%	497	21.3%	0.358
No	4,907	79.7%	1,195	80.4%	1,873	80.1%	1,839	78.7%	

* P values derived using Pearson chi-square test

Table S2. Sociodemographic and health characteristics by numbers of general practitioner (GP) consultations among adults diagnosed with lung cancer*

		GP consults						
		0-7		8-16		17 or more		
		n	%	n	%	n	%	p
Total		1,852	100.0%	2,651	100.0%	1,657	100.0%	
**Sociodemographic factors**								
Mean age (years)	n (95%CI)	66.8 (66.3-67.4)		69.0 (68.6-69.5)		71.6 (71.1-72.1)		<0.001
Sex								
	Males	1,056	57.0%	1,351	51.0%	869	52.4%	<0.001
	Females	796	43.0%	1,300	49.0%	788	47.6%	
Geographic remoteness								
	Major cities	1,164	62.9%	1,770	66.8%	1,186	71.6%	<0.001
	Inner regional	479	25.9%	645	24.3%	339	20.5%	
	Outer regional	209	11.3%	236	8.9%	132	8.0%	
Country of birth								
	Australia	1,213	65.5%	1,576	59.5%	960	57.9%	<0.001
	China	55	3.0%	72	2.7%	49	3.0%	
	Greece	15	0.8%	30	1.1%	33	2.0%	
	Italy	13	0.7%	62	2.3%	49	3.0%	
	Lebanon	24	1.3%	24	0.9%	31	1.9%	
	New Zealand	44	2.4%	59	2.2%	26	1.6%	
	the Philippines	10	0.5%	24	0.9%	13	0.8%	
	United Kingdom	159	8.6%	244	9.2%	150	9.1%	
	Vietnam	18	1.0%	34	1.3%	15	0.9%	
	Other English-speaking	61	3.3%	110	4.1%	45	2.7%	
	non-English speaking	240	13.0%	416	15.7%	286	17.3%	
Sole person household								
	Yes	489	26.4%	646	24.4%	438	26.4%	0.189
	No	1,363	73.6%	2,005	75.6%	1,219	73.6%	
**Socioeconomic factors**								
Poor English proficiency								
	Yes	99	5.3%	171	6.5%	156	9.4%	<0.001
	No	1,753	94.7%	2,480	93.5%	1,501	90.6%	
Low income								
	Yes	523	28.2%	881	33.2%	655	39.5%	<0.001
	No	1,329	71.8%	1,770	66.8%	1,002	60.5%	
Younger with disability								
	Yes	76	4.1%	151	5.7%	139	8.4%	<0.001
	No	1,776	95.9%	2,500	94.3%	1,518	91.6%	
Unemployed								
	Yes	48	2.6%	44	1.7%	17	1.0%	0.002
	No	1,804	97.4%	2,607	98.3%	1,640	99.0%	
Labourer								
	Yes	78	4.2%	92	3.5%	24	1.4%	0.000
	No	1,774	95.8%	2,559	96.5%	1,633	98.6%	
Machinery operator/driver								

Yes	57	3.1%	61	2.3%	25	1.5%	0.009
No	1,795	96.9%	2,590	97.7%	1,632	98.5%	
Less than Year 12 schooling attained							
Yes	795	42.9%	1,231	46.4%	829	50.0%	<0.001
No	1,057	57.1%	1,420	53.6%	828	50.0%	
No education							
Yes	32	1.7%	46	1.7%	51	3.1%	0.005
No	1,820	98.3%	2,605	98.3%	1,606	96.9%	
Renting from housing authority							
Yes	139	7.5%	218	8.2%	185	11.2%	<0.001
No	1,713	92.5%	2,433	91.8%	1,472	88.8%	
Jobless household with children							
Yes	35	1.9%	33	1.2%	30	1.8%	0.166
No	1,817	98.1%	2,618	98.8%	1,627	98.2%	
One parent with dependents							
Yes	27	1.5%	47	1.8%	28	1.7%	0.712
No	1,825	98.5%	2,604	98.2%	1,629	98.3%	
**Health factors**							
Numbers of conditions medicated (Rx Risk)							
0-2	832	44.9%	521	19.7%	134	8.1%	<0.001
3-5	709	38.3%	1,160	43.8%	468	28.2%	
6 or more	311	16.8%	970	36.6%	1,055	63.7%	
Local stage at diagnosis							
Yes	333	18.0%	535	20.2%	385	23.2%	<0.001
No	1,519	82.0%	2,116	79.8%	1,272	76.8%	

*P values derived using Pearson chi-square

Table S3. Sociodemographic and health characteristics by exposure to MBS-funded lung CT scans among adults diagnosed with lung cancer*

		CT of lung		No CT of the lung		
		n	%	n	%	P
Total		3,306	100.0%	2,854	100.0%	
**Sociodemographic characteristics**						
Mean age (years)	n (95%CI)	68.9 (68.6-69.3)		69.0 (68.8-69.3)		0.279
Sex						
	Males	1,733	52.4%	1,543	54.1%	0.197
	Females	1,573	47.6%	1,311	45.9%	
Geographic remoteness						
	Major cities & others	2,276	68.8%	1,844	64.6%	0.002
	Inner regional	739	22.4%	724	25.4%	
	Outer regional/remote	291	8.8%	286	10.0%	
Country of birth						
	Australia	1,958	59.3%	1,791	62.8%	0.003
	China	104	3.1%	72	2.5%	
	Greece	40	1.2%	38	1.3%	
	Italy	83	2.5%	41	1.4%	
	Lebanon	47	1.4%	32	1.1%	
	New Zealand	73	2.2%	56	2.0%	
	the Philippines	20	0.6%	27	0.9%	

United Kingdom	290	8.8%	263	9.2%	
Vietnam	46	1.4%	21	0.7%	
Other English	119	3.6%	97	3.4%	
non-English	526	15.9%	416	14.6%	
Sole person household					
Yes	797	24.1%	776	27.2%	0.006
No	2,509	75.9%	2,078	72.8%	
**Socioeconomic characteristics**					
Poor English proficiency					
Yes	247	7.5%	179	6.3%	0.064
No	3,059	92.5%	2,675	93.7%	
Low income (Household<$25,999)					
Yes	1,114	33.7%	945	33.1%	0.627
No	2,192	66.3%	1,909	66.9%	
Younger with disability (6 months and aged less than 70 years)					
Yes	166	5.0%	200	7.0%	0.001
No	3,140	95.0%	2,654	93.0%	
Unemployed					
Yes	50	1.5%	59	2.1%	0.100
No	3,256	98.5%	2,795	97.9%	
Labourer					
Yes	105	3.2%	89	3.1%	0.897
No	3,201	96.8%	2,765	96.9%	
Machinery operator & driver					
Yes	63	1.9%	80	2.8%	0.020
No	3,243	98.1%	2,774	97.2%	
Less than Year 12 schooling attained					
Yes	1,479	44.7%	1,376	48.2%	0.006
No	1,827	55.3%	1,478	51.8%	
No education					
Yes	55	1.7%	74	2.6%	0.011
No	3,251	98.3%	2,780	97.4%	
Renting from housing authority					
Yes	256	7.7%	286	10.0%	0.002
No	3,050	92.3%	2,568	90.0%	
Jobless household with children					
Yes	39	1.2%	59	2.1%	0.005
No	3,267	98.8%	2,795	97.9%	
One parent with dependents					
Yes	55	1.7%	47	1.6%	0.959
No	3,251	98.3%	2,807	98.4%	
**Health characteristics**					
Numbers of conditions medicated (Rx Risk)					
0-2	782	23.7%	705	24.7%	0.577
3-5	1,255	38.0%	1,082	37.9%	
6 or more	1,269	38.4%	1,067	37.4%	
GP consults					
0-7	785	23.7%	1,067	37.4%	<0.001
8-16	1,481	44.8%	1,170	41.0%	
17 or more	1,040	31.5%	617	21.6%	
Local stage at diagnosis					
Yes	870	26.3%	383	13.4%	<0.001
No	2,436	73.7%	2,471	86.6%	

*P values derived using Pearson chi square

Table S4. Sociodemographic and health characteristics by local stage at diagnosis among adults diagnosed with lung cancer*

		Extent of disease at diagnosis				
		Local stage		Regional/Distant/Unknown stage		
		n	%	n	%	p
Total		1,253	100.0%	4,907	100.0%	
**Sociodemographic factors**						
Mean age (years)	n (95%CI)	68.7 (68.0-69.2)		69.1 (68.8-69.53)		0.254
Sex						
	Males	582	46.4%	2,694	54.9%	<0.001
	Females	671	53.6%	2,213	45.1%	
Geographic remoteness						
	Major cities & others	841	67.1%	3,279	66.8%	0.433
	Inner regional	285	22.7%	1,178	24.0%	
	Outer regional/remote	127	10.1%	450	9.2%	
Country of birth						
	Australia	759	60.6%	2,990	61.0%	0.992
	China	39	3.1%	137	2.8%	
	Greece			Small counts suppressed		
	Italy	27	2.2%	97	2.0%	
	Lebanon			Small counts suppressed		
	New Zealand	27	2.2%	102	2.1%	
	the Philippines			Small counts suppressed		
	United Kingdom	116	9.3%	437	8.9%	
	Vietnam			Small counts suppressed		
	Other English	42	3.4%	174	3.5%	
	non-English	194	15.5%	748	15.2%	
Sole person household						
	Yes	319	25.5%	1,254	25.6%	0.944
	No	934	74.5%	3,653	74.4%	
**Socioeconomic factors**						
Poor English proficiency						
	Yes	98	7.8%	328	6.7%	0.157
	No	1,155	92.2%	4,579	93.3%	
Low income (Household<$25,999)						
	Yes	417	33.3%	1,642	33.5%	0.903
	No	836	66.7%	3,265	66.5%	
Younger with disability (6 months and aged less than 70 years)						
	Yes	63	5.0%	303	6.2%	0.125
	No	1,190	95.0%	4,604	93.8%	
Unemployed						
	Yes	16	1.3%	93	1.9%	0.138
	No	1,237	98.7%	4,814	98.1%	
Labourer						
	Yes	32	2.6%	162	3.3%	0.176
	No	1,221	97.4%	4,745	96.7%	
Machinery operator & driver						
	Yes	26	2.1%	117	2.4%	0.516
	No	1,227	97.9%	4,790	97.6%	

Less than Year 12 schooling attained						
	Yes	560	44.7%	2,295	46.8%	0.188
	No	693	55.3%	2,612	53.2%	
No education						
	Yes	22	1.8%	107	2.2%	0.349
	No	1,231	98.2%	4,800	97.8%	
Renting from housing authority						
	Yes	108	8.6%	434	8.8%	0.802
	No	1,145	91.4%	4,473	91.2%	
Jobless household with children						
	Yes	22	0.5%	76	1.8%	<0.001
	No	1,231	99.5%	4,831	98.2%	
One parent with dependents						
	Yes	20	1.6%	82	1.7%	0.853
	No	1,233	98.4%	4,825	98.3%	
**Health factors**						
Numbers of conditions medicated (Rx Risk)						
	0-2	292	23.3%	1,195	24.4%	0.358
	3-5	464	37.0%	1,873	38.2%	
	6 or more	497	39.7%	1,839	37.5%	
GP consults						
	0-7	333	26.6%	1,519	31.0%	0.001
	8-16	535	42.7%	2,116	43.1%	
	17 or more	385	30.7%	1,272	25.9%	

*P values derived using Pearson chi-square
